# Photochemistry of 1-Phenyl-1-diazopropane and Its Diazirine Isomer: A CASSCF and MS-CASPT2 Study

**DOI:** 10.1021/acs.jpca.2c04816

**Published:** 2022-11-06

**Authors:** Juan Soto

**Affiliations:** Department of Physical Chemistry, Faculty of Science, University of Málaga, 29071 Málaga, Spain

## Abstract

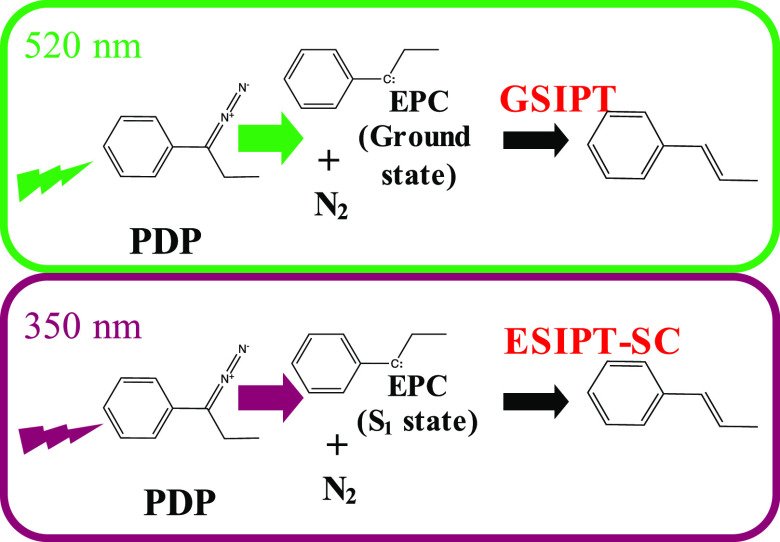

In this work, we studied the wavelength (520 or 350 nm) dependence of the photochemical decomposition of 1-phenyl-1-diazopropane (**PDP**) and 1-phenyl-1-propyl diazirine (**PED**) by means of high-level *ab initio* quantum chemical calculations (CASSCF and MS-CASPT2) to obtain qualitative and quantitative results. It is found that the photochemistry of **PDP** is governed by nonradiative deactivation processes that can involve one or two S_1_/S_0_ conical intersections (**CI1** and **CI2**) depending on the wavelength of the radiation; **CI2** is only accessible at the shortest wavelength. It is demonstrated that the main intermediate of the photochemistry of the titled compounds is 1-ethyl-1-phenyl carbene (**EPC**). Upon irradiation of **PDP** with the 520 nm light, the carbene is always generated in its ground state as closed-shell singlet carbene. In contrast, the 350 nm radiation can directly decompose **PDP** into S_1_ carbene (open shell) and N_2_ when the conical intersection **CI2** is avoided. Once the carbene is formed in the S_1_ state, it can experience excited state intramolecular proton transfer along a seam of crossing (ESIPT-SC) of the S_1_ and S_0_ states to yield the alkene derivative; that is, the proton transfer reaction takes places on a degenerate potential energy surface where the two electronic states have equal energy. In addition, it is found that **EPC** absorbs at 350 nm (double excitations); therefore, there is another possible route that can induce as well a slightly different photochemistry in changing the wavelength of the radiation because the shortest wavelength (when it is intense enough) decreases the amount of available **EPC** or generates a highly vibrationally excited state of the carbene; that is, after 350 nm excitation, the carbene intermediate can deactivate via radiation emission or can decay through a cascade of conical intersections to its first excited state (S_1_), where ESIPT-SC is operative again.

## Introduction

Carbenes are chemical species that play important roles as intermediates in many organic synthetic routes.^1−10^ Such species have a rich and variable chemistry due to the divalent carbon atoms that this class of molecules bears, given that such an atom can be electronically configured as an open shell (singlet or triplet) or a closed shell (singlet). The spin of the ground state of the carbene depends strongly on the substituents of the divalent carbon.^9^

Carbenes can be produced upon photolysis of ketones, diazo compounds, or diazirines.^10−19^ Curiously, while diazo compounds have been used in synthetic chemistry, their structural isomers (diazirines) have been reserved to other applications as photolabeling reagents of proteins^20,21^ or polymer cross-linking.^10^ The trend with diazirines has changed only recently.^10,19^

With regard to the spin and electronic states of carbene photolytically formed, Prof. Platz et al. have recently published an article^22^ within a special issue in honor of Prof. Carpenter in which it is found that the photolysis of 1-phenyl-1-diazopropane (**PDP**) depends on the irradiating wavelength. To be specific, irradiation at 520 nm yields the closed-shell singlet carbene intermediate (1-ethyl-1-phenyl carbene (**EPC**)) that isomerizes to 1-phenylpropene, or it is trapped with methanol to form the corresponding ether. In contrast, it is proposed that irradiation at 350 nm generates two classes of carbene intermediates: one that can be captured with methanol (S_0_ closed-shell carbene) and the other that cannot be (open-shell carbene).

**Scheme 1:**
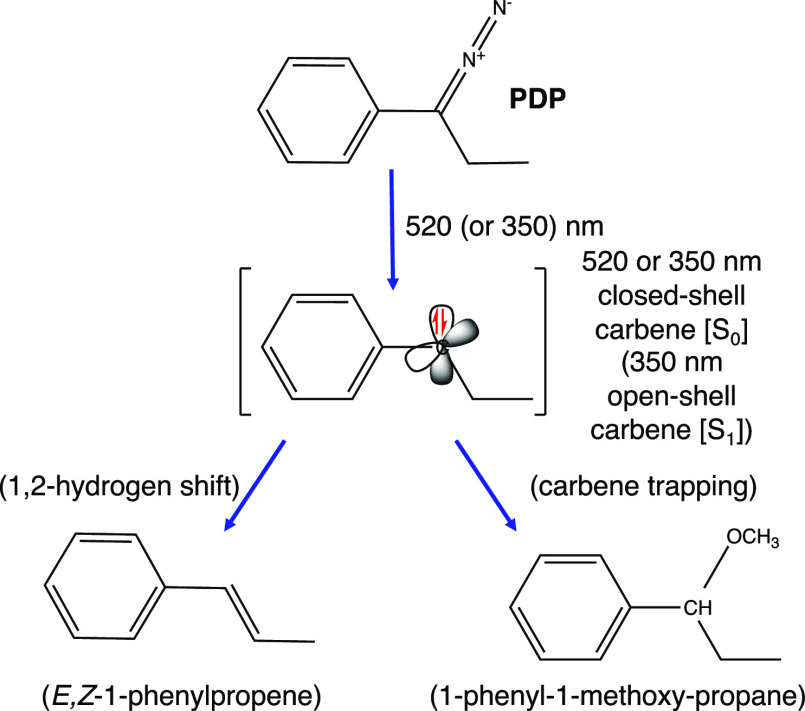
Main Reaction Paths of the Photochemistry of PDP

From the theoretical point of view, the conclusions reported by the former authors are very attractive and even more when it is found that, as will be shown in this text, independently of the wavelength applied to the system (520 or 350 nm), only the first excited state of the diazo compound (**PDP**) is populated, that is, S_1_. Thus, herein, we report the computational study of the photochemical decomposition of the title molecule and its diazirine isomer, 1-phenyl-1-propyl diazirine (**PED**), which are two intimately related systems both electronically and chemically. To this end, we have performed *ab initio* quantum chemical calculations at the CASSCF and MS-CASPT2 levels, which are one of the most appropriate theoretical approximations to deal with this class of molecules and most importantly this type of reactions.^23−26^ It will be shown that our work corroborates and explains in detail the assertions and conclusions given by Platz et al.,^22^ excepting the population of the second excited state of the diazo compound upon irradiation with the 350 nm light. In fact, we will demonstrate that the variation of the reactivity in changing the excitation wavelength is due to the absorption of the intermediate carbene (**EPC**) at 350 nm. The mechanism proposed in this work for the photochemistry of **PDP** is sketched in Scheme 1.

## Results and Discussion

In accordance with the objectives of this work, the manuscript is organized as follows: First, we identify and select the most relevant orbitals that are involved both in the electronic excitations and the traveling of the molecules along the reactive potential energy surfaces of the ground and low-lying excited states. These orbitals will define what is called the active space, that is, the orbitals that will be doubly optimized (optimizations of molecular orbitals and configuration interaction coefficients). This step is crucial in any CASSCF study and will determine the quality of our results and conclusions.^27−30^ Second, calculation of the electronic energies of the excited states of the diazo and diazirine compounds to determine the electronic states accessible at the wavelengths used in the experiments of Platz and co-workers. Third and fourth, exploration of the potential energy surfaces for searching the critical points that lead to diazo ↔ diazirine isomerization or denitrogenation and formation of carbenes from the diazo precursor. Fifth, a 1,2-hydrogen shift in the carbene intermediate to yield the *E*(*Z*)-ethylene derivatives. Sixth, conclusions. Seventh, computational details.

### Selection of the Active Spaces of Diazo and Diazirine Derivatives

**Figure 1:**
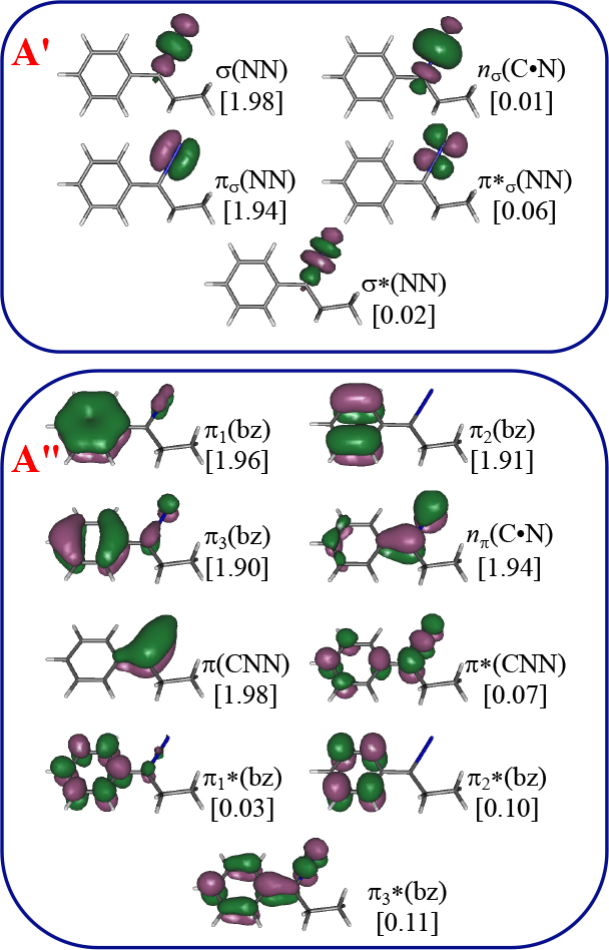
CASSCF/ANO-RCC natural orbitals included in the active space (14e, 14o) of PDP. Ground state CASSCF optimized geometry. In square brackets: occupation numbers.

**Figure 2:**
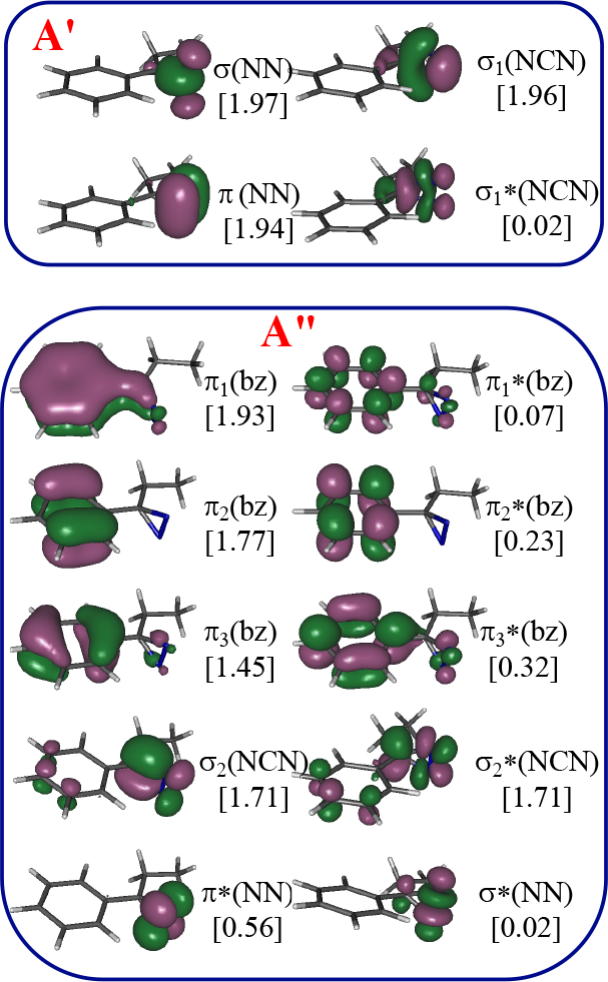
CASSCF/ANO-RCC natural orbitals included in the active space (14e, 14o) of PED. Ground state CASSCF optimized geometry. In square brackets: occupation numbers.

As mentioned before, the selection of the active space is crucial because an inappropriate active space could lead to erroneous results.^27−30^ In this work, we have taken advantage of previous studies on diazirine and diazo compounds.^12,31,32^ Thus, the active spaces of the diazo and diazirine compounds studied in this work comprise eight orbitals and eight electrons that arise from the six σ-orbitals and the two π-orbitals of the diazo(diazirine) moiety plus the six π-type orbitals of the phenyl substituent. Therefore, the total size of the active space is 14 electrons distributed in 14 orbitals. Pictorial representations and detailed descriptions of such orbitals are given in Figures 1 and 2 for diazo and diazirine derivatives, respectively.

### Energetics of the Singlet and Triplet States at the Franck–Condon Geometry of the Diazo and Diazirine Compounds

The calculated energies in eV of the low-lying singlet and triplet vertical excitations of **PDP** and **PED** are collected in Tables 1 and 2, respectively, where the character and oscillator strengths of the electronic transitions are included as well. These results agree with previous data obtained by Platz et al.^33^ According to the data reported in Table 1, absorption of photons by **PDP** at 520 nm (2.38 eV) is not energetically allowed. However, after correcting for the zero-point vibrational energy of the S_0_, S_1_, and S_2_ states, we found that the 0–0 transitions of the two lowest singlet excited states of the diazo compound are localized at 1.70 and 4.3 eV, respectively. Therefore, S_1_ is the only accessible state with the wavelengths of 520 (2.38) or 350 (3.54 eV) nm.

**Table 1:** Vertical Excitation Energies (Δ*E*) in eV of the Singlet and Triplet States of 1-Phenyl-1-diazopropane (**PDP**)^a^^,^^b^

state	Δ*E*	excitation^c^	*f*_OSC_^d^	configuration^e^	*W*^f^
1^1^A″	2.59	π → π*	1.25 × 10^–05^	[π_3_]^1^[π_3_*]^1^	78
2^1^A′	4.31	π → π*	3.33 × 10^–01^	[π_3_]^1^[π_3_*]^1^	43
				[π_3_]^1^[π_2_*]^1^	17
3^1^A′	4.58	π → π*	2.83 × 10^–01^	[π_2_]^1^[π_3_*]^1^	18
				[π_3_]^1^[π_3_*]^1^	30
				[π_3_]^1^[π_2_*]^1^	19
2^1^A″	5.56	π → π*	2.14 × 10^–05^	[π_2_]^1^[π_σ_*]^1^	44
				[π_2_]^0^[π_3_*]^1^[π_σ_*]^1^	25
3^1^A″	5.64	π → π*		[π_2_]^1^[π_σ_*]^1^	47
				[*n*_π_]^0^[π_3_*]^1^[π_σ_*]^1^	19
4^1^A′	6.20	*n* → π*	1.52 × 10^–03^	[*n*_π_]^1^[π_3_*]^1^	21
		π → π*		[π_3_]^1^[π*(CNN)]^1^	15
		π → π*		[π_3_]^0^[π_3_*]^2^	32
4^1^A″	7.21	*n* → π*		[*n*_π_]^1^[π_2_]^1^[π_2_*]^1^[π_σ_*]^1^	57
1^3^A′	3.07	π → π*		[π_3_]^1^[π_3_*]^1^	67
2^3^A′	4.35	π → π*		[π_2_]^1^[π_3_*]^1^	21
		π → π*		[π_3_]^1^[π_2_*]^1^	48
1^3^A″	2.42	*n* → π*		[*n*_π_]^1^[π_σ_*]^1^	76
2^3^A″	5.53	π → π*		[π_2_]^1^[π_σ_*]^1^	42

a *C*_s_ MP2/def2-TZVPP optimized geometry.

b SA4-CASSCF reference wave function, IPEA = 0.25. Imaginary shift = 0.1.

c Character of the excitation.

d Oscillator strength.

e MS-CASPT2 main electronic configurations of the excited states referred to the ground state configuration.

f Weight of the configuration in %. Only contributions greater than 15% are included.

**Table 2:** Vertical Excitation Energies (Δ*E*) in eV of the Singlet and Triplet States of 1-Phenyl-1-propyl Diazirine (**PED**)^a^^,^^b^

state	Δ*E*	excitation^c^	*f*_OSC_^d^	configuration^e^	*W*^f^
2^1^A′	3.53	π → π*	4.46 × 10^–3^	[π_3_]^1^[π*(NN)]^1^	64
3^1^A′	4.84	π → π*	5.29 × 10^–5^	[π_3_]^1^[π*(NN)]^1^	28
				[π_3_]^1^[π_2_*]^1^	21
4^1^A′	5.96	σ → π*	1.17 × 10^–3^	[σ_2_]^1^[π*(NN)]^1^	34
				[π_3_]^0^[π_3_*]^1^[π*(NN)]^1^	16
1^1^A″	7.66	σ → π*	<10^–5^	[σ_1_]^1^[π*(NN)]^1^	76
2^1^A″	8.89	π → π*	7.70 × 10^–4^	[π(NN)]^1^[π_3_]^1^[π*(NN)]^1^[π_3_*]^1^	28
3^1^A″	9.41	π → π*	7.31 × 10^–3^	[π(NN)]^1^[π_3_]^1^[π*(NN)]^1^[π_2_*]^1^	15
4^1^A″	9.50	π → π*	1.30 × 10^–4^	[π(NN)]^1^[π_3_]^1^[π*(NN)]^1^[π_2_*]^1^	19
1^3^A′	3.10	π → π*		[π_3_]^1^[π*(NN)]^1^^1^	30
				[σ_2_]^1^[π*(NN)]	27
2^3^A′	4.07	π → π*		[π_2_]^1^[π_3_*]^1^	16
				[π_3_]^1^[π_2_*]^1^	33
1^3^A″	4.97	π → π*		[π(NN)]^1^[π*(NN)]^1^	83
2^3^A″	6.25	σ → π*		[σ(NN)]^1^[π*(NN)]^1^	82

a *C*_s_ MP2/def2-TZVPP optimized geometry.

b SA4-CASSCF reference wave function, IPEA = 0.25. Imaginary shift = 0.1.

c Character of the excitation.

d Oscillator strength.

e MS-CASPT2 main electronic configurations of the excited states referred to the ground state configuration.

f Weight of the configuration in %. Only contributions greater than 15% are included.

Concerning the diazirine derivative, according to the data of Table 2, it could be possible that this molecule was excited to S_1_ with the shortest wavelength at 350 nm, which corresponds to the 2^1^A′ state. In any case, it will be shown in the next sections that diazirine is a short-lived intermediate in the photolysis of **PDP**, which rapidly decomposes to yield the carbene intermediate in its lowest singlet state (closed-shell) and molecular nitrogen.

### Photochemistry of the Diazo and Diazirine Compounds (PDP and PED)

**Figure 3:**
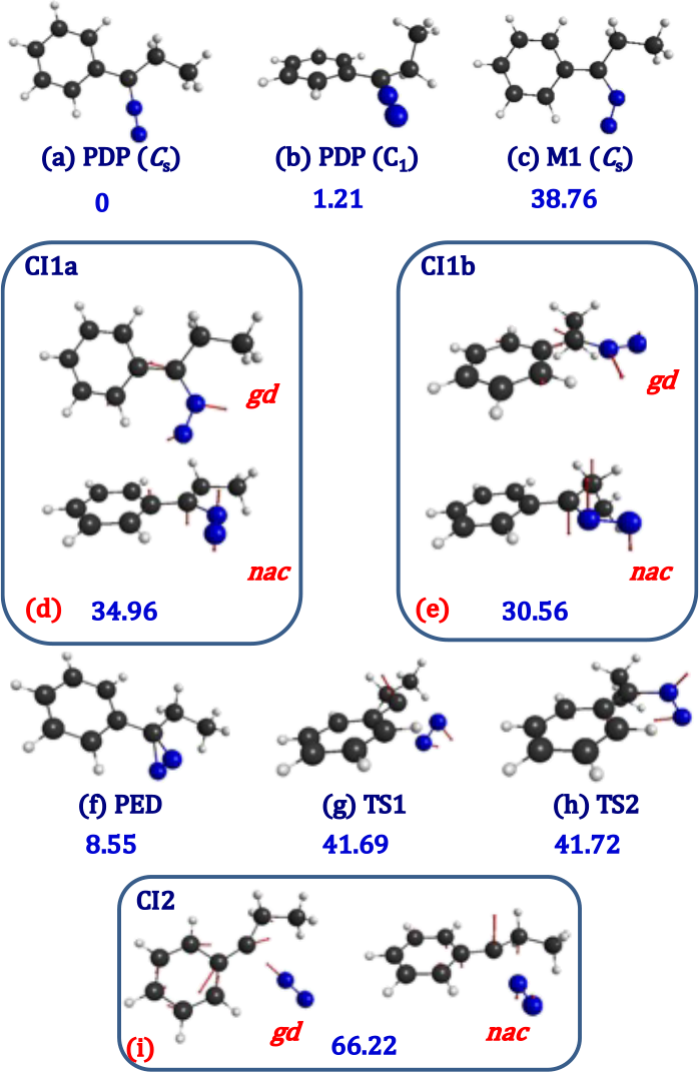
CASSCF(14e, 14o)/ANO-RCC optimized geometries. (a) 1-phenyl-1-diazopropane [*C*_s_, S_0_]; (b) 1-phenyl-1-diazopropane [*C*_1_, S_0_]; (c) 1-phenyl-1-diazopropane [S_1_]; (d) S_1_/S_0_ conical intersection for diazo–diazirine isomerization; (e) S_1_/S_0_ conical intersection for diazo–diazirine isomerization (from conformer *C*_1_); (f) 3-phenyl-3-ethyl-3*H*-diazirine [S_0_]; (g) transition state for N_2_ extrusion [S_0_]; (h) transition state for diazo–diazirine isomerization [S_0_]; and (i) S_1_/S_0_ conical intersection or N_2_ extrusion. **gd**: gradient difference vector. **nac**: nonadiabatic coupling vector. In blue numbers: relative electronic energies with respect to 1-phenyl-1-diazopropane *C*_s_ [S_0_] in kcal/mol (SA2-MS-CASPT2).

**PDP** has two stable conformers on the S_0_ ground state surface, one has *C*_s_ symmetry and the other belongs to the *C*_1_ symmetry point group (Figure 3a,b). In what follows, for computational convenience, we will restrict our discussion to the *C*_s_ conformer. Given that only the S_1_ excited state can be reached with the two wavelengths used in the experiments, we will focus our attention on the reactivity on the S_1_ potential energy surface of **PDP**. It is found that there exist the following relevant critical points that are relevant in the photochemistry of the molecule starting at the S_1_ state: (i) A minimum energy point **M1** (Figure 3c), which is localized at 16.22 kcal/mol below the 520 nm excitation light. (ii) Two S_1_/S_0_ conical intersections, **CI1a** (Figure 3d) and **CI1b** (Figure 3e), which lie 20.02 and 24.42 kcal/mol below the 520 nm excitation, respectively. These two conical intersections connect the S_1_ state of **PDP** with the S_0_ state of 3-phenyl-3-ethyl-3*H*-diazirine (**PED**; Figure 3f), as demonstrated in previous works.^31,32^**CI1a** is associated to the *C*_s_ conformer and **CI1b** to the *C*_1_ one. (iii) Another S_1_/S_0_ conical intersection, **CI2** (Figure 3g), which lies 11.14 kcal/mol above the 520 nm excitation and leads to direct denitrogenation of the diazo compound. This third conical intersection (**CI2**) is only accessible upon irradiation with the 350 nm light. Our results differ slightly with those ones obtained by Zhang et al.,^33^ who found a transition state connecting the S_1_ minima of phenyldiazirine and phenyldiazomethane.

Concerning the photochemistry upon irradiation with 520 nm, once the Franck–Condon point is populated with the excitation energy at 520 nm, the system decays to a minimum on the S_1_ surface (**M1**; Figure 3c). Then, via an almost barrierless process, the molecule deactivates to the S_0_ state through a S_1_/S_0_ conical intersection (**CI1a**; Figure 3d) that leads to diazo–diazirine isomerization (Figure S1). The analogous conical intersection exists for the *C*_1_ conformer (**CI1b**; Figure 3e). Regardless of whether the system starts from the *C*_s_ or *C*_1_ conformer, we do not expect the formation of the diazirine compound (**PED**; Figure 3f) as a photoproduct because it arises from a rapid nonradiative deactivation process that conserves all the photonic excitation energy as internal energy. In contrast, **PED** will dissociate into carbene in its closed-shell ground state and molecular nitrogen through the transition state **TS1** represented in Figure 3g. In fact, this is what we observed in our dynamics study on diazomethane.^32^ In addition, there exists another alternative route after reaching the domain of diazirine on the S_0_ state via the nonradiative deactivation through the S_1_/S_0_ conical intersection, that is, diazirine–diazo isomerization through the transition state **TS2** (Figure 3h), but this channel will again end as dissociation into S_0_ carbene and N_2_.

With respect to the chemical processes originated from irradiation with the 350 nm light, as was established in previous paragraphs, again, only the S_1_ state is reached with this wavelength. Therefore, we expect that the same reaction mechanisms operating with the 520 nm light will be active with the shortest wavelength as well. In addition, we have found an additional channel that leads to direct formation of the carbene intermediate, which is not accessible with 520 nm. Again, this new channel starts at the Franck–Condon point and passes through a S_1_/S_0_ conical intersection (**CI2**; Figure 3i), which lies 15.47 kcal/mol below the excitation wavelength at 350 nm. The carbene intermediate would be generated in its S_0_ ground state (closed shell) if the surface crossing process occurs.

However, given that the parent diazo compound has been generated in a highly vibrational excitation at 350 nm, the system can avoid the surface hop to dissociate into N_2_ and carbene in its S_1_ excited state. This open-shell singlet carbene (S_1_) can experience S_1_/S_0_ surface crossing through **CI4** (Figure 6e) or **CI5** (Figure 6l) conical intersections or 1,2-H rearrangement (see Section 5). The energy profiles leading to direct dissociation of the diazo compound are represented in Figure S2, where it is clearly observed that the **CI2** crossing is a sloped conical intersection, which suggests that the surface hop can be easily avoided.

### Ground State 1,2-Hydrogen Shift in the Carbene–Ethylene Rearrangement

**Figure 4:**
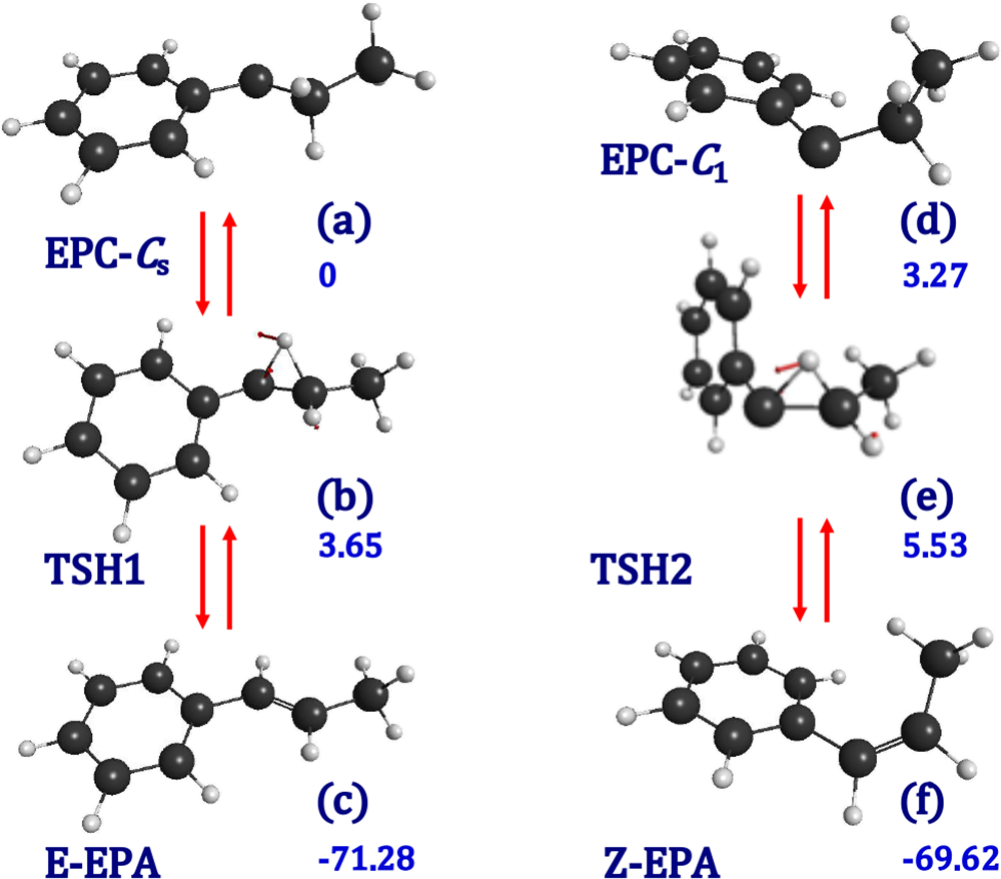
CASSCF(10e, 10o)/ANO-RCC optimized geometries of the species involved in the 1,2-hydrogen migration: (a) 1-ethyl-1-phenyl carbene (*C*_s_); (b) transition state for carbene → *E*-alkene isomerization; (c) *E*-1-phenylpropene; (d) 1-ethyl-1-phenyl carbene (*C*_1_); (e) transition state for carbene → *Z*-alkene isomerization; and (f) *Z*-1-phenylpropene. In blue numbers: relative electronic energies with respect to 1-ethyl-1-phenyl carbene (*C*_s_) in kcal/mol (CASPT2).

On the ground state surface, isomerization of the closed-shell carbene intermediate to *E*- or *Z*-1-phenylpropene is a highly exothermic process that requires a very low activation energy (roughly ∼2–3 kcal/mol). This value of the activation energy contrasts with the values reported by other authors (∼7 kcal/mol) for 1,2-hydrogen migration of dialkylcarbenes.^34,35^Figure 4 displays the geometries of the reactants, products, and transition states that are involved in 1,2 hydrogen migration to form *E*- and *Z-*1-phenylpropene, respectively, along with the relative electronic energies of each of the species. Formation of *E*- or *Z*-alkenes depends on the initial conformation of the reactive carbene (*C*_s_ or *C*_1_ conformer). *C*_s_ carbene (Figure 4a) with orientation of the pair of free electrons in opposition to the hydrogen atoms of the −CH_2_– moiety yields *E*-1-phenylpropene, whereas *C*_1_ carbene (Figure 4e), which has the pair of free electrons oriented in the same direction as that of the two hydrogen atoms of the −CH_2_– moiety, forms *Z*-1-phenylpropene.

### Vertical Excitation Energies of the Carbene Intermediate. Excited State Intramolecular Proton Transfer along the Seam of Crossing (ESIPT-SC) of the S_1_/S_0_ States

It has been shown in the previous section that the parent molecule (**PDP**) does not reach a higher excited state than S_1_ when irradiated at 350 nm. In this section, we have investigated the excitation properties of the carbene intermediate and excited state intramolecular proton transfer (ESIPT)^36−40^ or rearrangements in excited states (RIES).^41^

**Figure 5:**
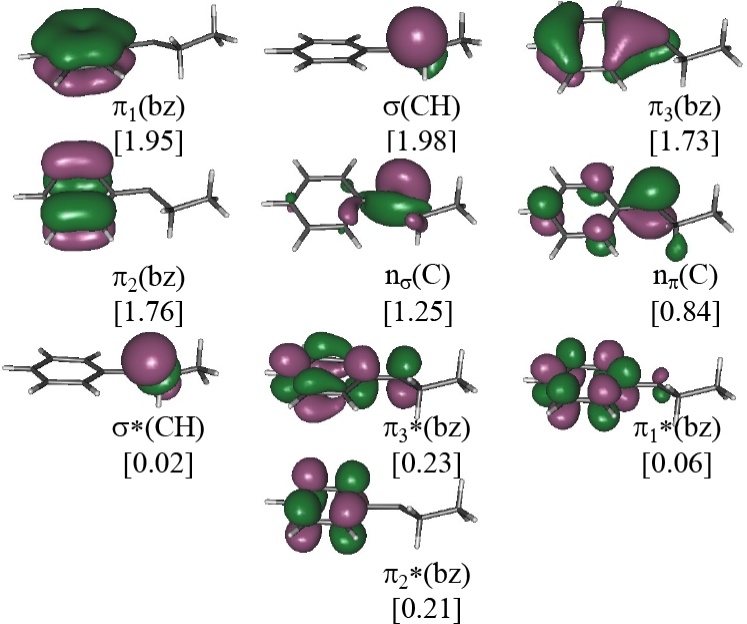
CASSCF/ANO-RCC natural orbitals included in the active space (10e, 10o) of *E*-1-ethyl-1-phenyl carbene. Ground state CASSCF optimized geometry. In square brackets: occupation numbers.

The vertical excitation energies of both conformers of the carbene intermediate are collected in Table 3. To clarify the assignment given in Table 3, the orbitals included in the active space of the *E*-conformer are represented in Figure 5.

**Table 3:** Vertical Excitation Energies (Δ*E*) in eV of the Singlet States of *E*,*Z*-1-Ethyl-1-phenyl Carbene^a^^,^^b^

state	Δ*E*	excitation^c^	*f*_OSC_^d^	configuration^e^	*W*^f^
*C*_s_ Conformer (*E*)					
1A′				[*n*_σ_(C)]^2^[*n*_π_(C)]^0^	78
2^1^A′	1.16	*n*_σ_ → *n*_π_	4.21 × 10^–3^	[*n*_σ_(C)]^1^[*n*_π_(C)]^1^	73
3^1^A′	3.46	*n*_σ_ → π*^g^	4.56 × 10^–4^	[*n*_σ_(C)]^1^[π_2_*]^1^	17
				[π_2_]^1^[*n*_σ_(C)]^1^[*n*_π_(C)]^2^	27
4^1^A′	3.54	*n*_σ_ → π*^g^	3.19 × 10^–3^	[*n*_σ_(C)]^1^[π_3_*]^1^	17
				[π_3_]^1^[*n*_σ_(C)]^1^[*n*_π_(C)]^2^	28
*C*_1_ Conformer (*Z*)					
1A′				[*n*_σ_(C)]^2^[*n*_π_(C)]^0^	83
2^1^A′	1.47	*n*_σ_ → *n*_π_	9.75 × 10^–3^	[*n*_σ_(C)]^1^[*n*_π_(C)]^1^	68
3^1^A′	3.29	*n*_σ_ → π*^g^	2.40 × 10^–3^	[*n*_σ_(C)]^1^[π_2_*]^1^^2^	15
				[*n*_σ_(C)]^0^[*n*_π_(C)]	21
4^1^A′	3.48	*n*_σ_ → π*^g^	6.11 × 10^–4^	[*n*_σ_(C)]^1^[π_3_*]^1^	15
				[*n*_σ_(C)]^0^[*n*_π_(C)]^2^	21

a CASSCF(10,10)/ANO-RCC optimized geometry.

b SA4-CASSCF(10,10) reference wave function, IPEA = 0.25. Imaginary shift = 0.1.

c Character of the excitation.

d Oscillator strength.

e MS-CASPT2 main electronic configurations of the excited states referred to the ground state configuration.

f Weight of the configuration in %. Only contributions greater than 15% are included.

g Double excitation.

**Figure 6:**
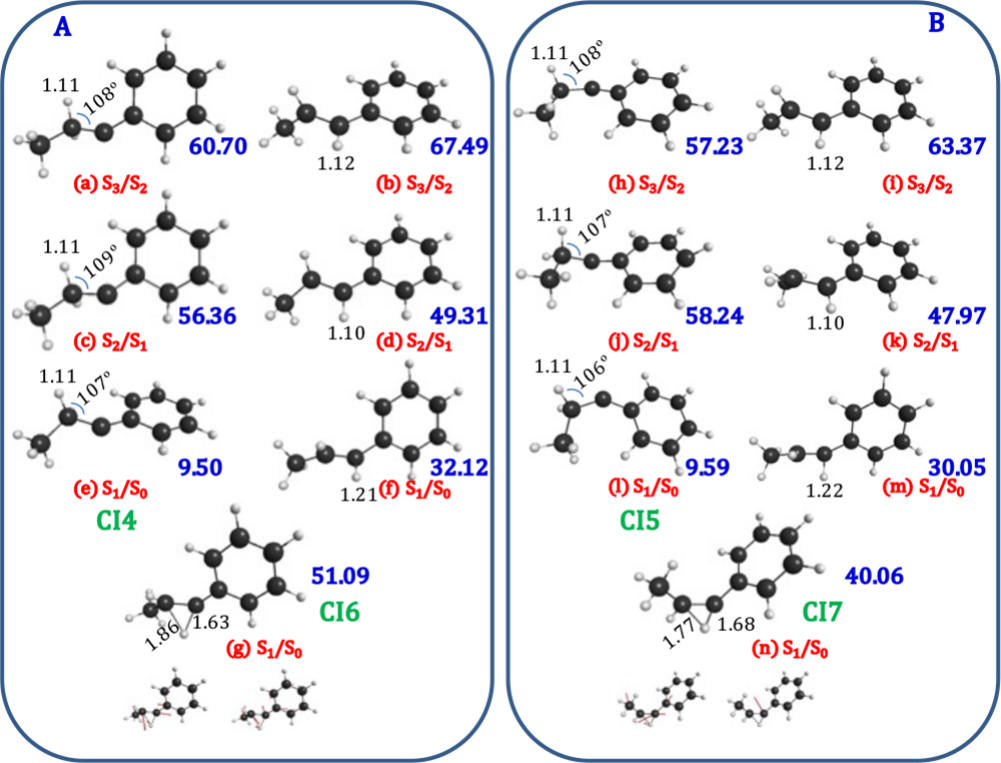
SA4-CASSCF(10,10)/ANO-RCC optimized conical intersection geometries of *C*_s_-carbenes (*E*-alkenes) (A) and *C*_1_-carbenes (Z-alkenes) (B). Figures (g) and (n) represent the transition state structures on the S_1_/S_0_ conical intersections. In blue numbers: relative energies of the structures with respect to the ground state of the *C*_s_ or *C*_1_ carbenes (SA4-MS-CASPT2).

Interestingly, it is found that there are two excited states (S_2_ and S_3_) accessible to both conformers when they are irradiated with the 350 nm wavelength light. In consequence, excitation at 350 nm would lead to a decrease in the reagent available to be trapped by methanol, as observed in the experiments of Platz et al., probably due to the following mechanism: after excitation of the carbene with 350 nm light, this intermediate can deactivate via radiation emission or can decay through a cascade of conical intersections to its first excited state (S_1_), and it experiences excited state intramolecular proton transfer along a S_1_/S_0_ seam of crossing (ESIPT-SC) to give the alkene derivative, as was postulated in Luk’s thesis,^42^ where it was found that a conical intersection connected excited open-shell singlet of 2-butylidene with butene. In fact, at this point, it must be noted that conical intersections are not isolated singularities on the potential energy surfaces of the molecules but rather are part of a seam of molecular rearrangements where the energy varies while keeping the degeneracy of the electronic states.^43^ In consequence, we can find as minima as transition states along the seam of crossing of the two degenerate states. To our concern, the geometries of the conical intersections corresponding to minimum energy points for both the *C*_s_,*C*_1_-carbenes and *Z*,*E*-alkenes, which would be accessible after 350 nm excitation, are represented in Figure 6. In addition, Figure 6g,n depicts the geometries of the degenerate transition states (**CI6** and **CI7**) on the S_1_/S_0_ seam of crossing that would lead to a 1,2-hydrogen shift from *C_s_* and *C*_1_ carbenes, respectively. ESIPT-SC at higher seams (S_2_/S_1_ or S_3_/S_1_) are not probable because the corresponding transition states would be localized at energies close to the excitation light or higher; for example, the degenerate transition state for isomerization on the *E*-S_1_/S_0_ seam (not included in Figure 6) is computed at 77 kcal/mol.

The mechanism proposed in the previous paragraph for ESIPT-SC starting at the S_3_ state of the carbene would be valid for an intense source of radiation. However, under the conditions that the experiments of Platz et al. are performed, it would not lead to a secondary photochemistry of carbene because the carbene produced with the UV lamp used in such experiments will always be present in a very low concentration, too low to absorb any light.

If this is the case, we must assume that carbene is formed in its S_1_ excited state through the mechanism that avoids the **CI2** conical intersection; in such a case, the 1,2-hydrogen shift (ESIPT-SC) would still occur through the **CI6** or **CI7** degenerate transition states, depending on the geometrical conformation of **PDP**. Notably, the barrier height of the **CI6** conical intersection (leading to *E-*alkene) is 11 kcal/mol higher than the barrier of **CI7** (leading to *Z*-alkene), which could be the reason for the increase of the *Z*/*E* ratio of the alkenes observed with the shorter wavelength.^22^

## Conclusions

In this work, we studied the reaction mechanism of the photochemical decomposition (at 520 and 350 nm, respectively) of 1-phenyl-1-diazopropane (**PDP**) and the excitation properties of its diazirine isomer, that is, 1-phenyl-1-propyl diazirine (**PED**). To this end, we have performed *ab initio* multiconfigurational calculations to reach qualitative and quantitative accuracy.^44,45^

**Scheme 2:**
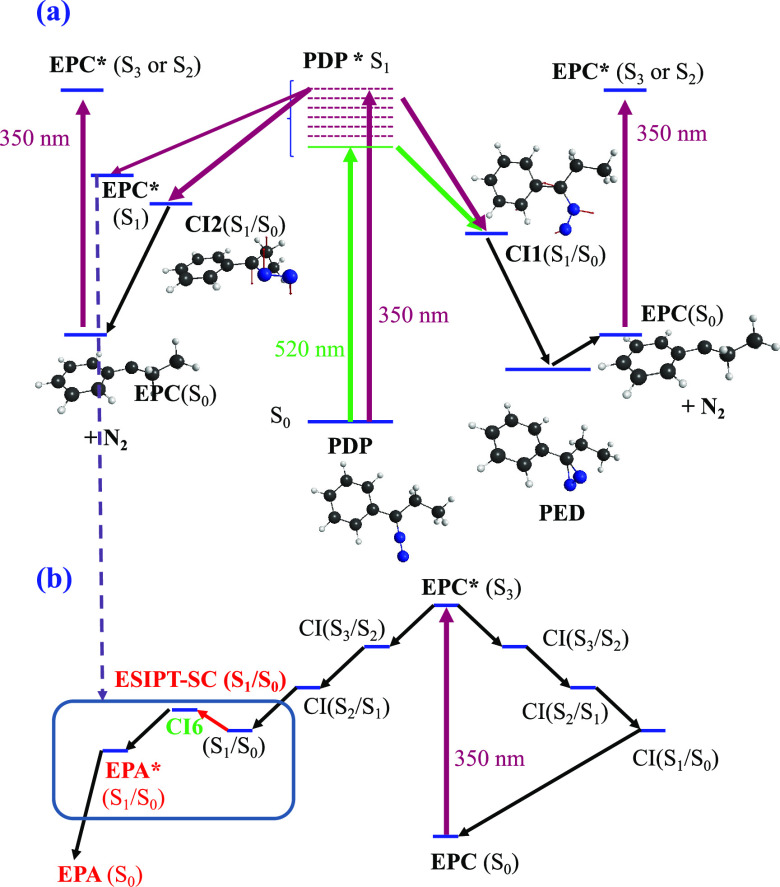
(a) Photochemical Mechanisms of the Decomposition of 1-Phenyl-1-diazopropane upon Irradiation with 520 or 350 nm Light and (b) Photochemistry of the Carbene Intermediates with Irradiation upon 350 nm

The photochemistry of PDP and its carbene product is represented in Scheme 2. The photochemical mechanism of **PDP** at 520 nm (excitation to the S_1_ state) is governed by a nonradiative deactivation process, which passes through a S_1_/S_0_ conical intersection (**CI1a** or **CI1b**; Figure 3d,e). After deactivation via this conical intersection, the potential well of the diazirine is sampled, but given that the molecule has accumulated a high amount of internal energy, which decomposes rapidly into molecular nitrogen and the carbene intermediate in its closed-shell electronic configuration, the same reaction path was observed by us on diazomethane by means of dynamical calculations.^32^ On the other hand, irradiation of **PDP** with 350 nm light again excites the compound to its S_1_ state, as occurs with the 520 nm light; however, there exists another energetically accessible channel, which leads as well to the formation of carbene and N_2_ via two different channels: (i) S_0_ carbene and N_2_ via S_1_/S_0_ surface crossing through the **CI2** conical intersection (Figure 3i) or (ii) S_1_ carbene plus N_2_ when the S_1_/S_0_ surface hop is avoided. Dissociation of **PDP** in the S_1_ state avoiding the nonradiative deactivation through **CI2** is a probable event for two reasons: (i) the excitation with the 350 nm wavelength adds an amount of internal energy well above the minimum crossing point **CI2**; (ii) the sloped topology of **CI2** favors to avoid the crossing (Scheme 2a). Carbene formed in its S_1_ state can rapidly decay to its ground state through a S_1_/S_0_ conical intersection (Figure 6e,l) or yields the alkene derivative via excited state intramolecular proton transfer along the seam of crossing (ESIPT-SC) formed by the degeneracy of the S_1_ and S_0_ states that connects the carbene–alkene system (Figure 6), whose electronic degenerate transition states are represented in Figure 6g,n for the *C*_s_ and *C*_1_ carbenes, respectively. Alternately, irradiation of the carbene intermediate with the 350 nm light can excite this intermediate to the S_3_ state and after a cascade of nonradiative transition ends at the S_1_ state, where ESIPT-SC is again operative (Scheme 2b).

## Computational Details

To achieve qualitative and quantitative accuracy,^44,45^ calculations have been performed with the complete active space self-consistent field (CASSCF)^46−50^ and the multistate second-order perturbation (MS-CASPT2)^51,52^ methods as implemented in MOLCAS 8.4.^53,54^ To avoid the inclusion of intruder states in the calculations, MS-CASPT2 energies have been calculated with an imaginary shift set to 0.1. IPEA empirical correction has been fixed at the standard value (0.25) in all of the calculations. CASSCF applied with the state average approximation is noted as SA*n*-CASSCF, where *n* refers to the number of states of a given symmetry species. The ANO-RCC basis sets^55−57^ have been used in the multiconfigurational calculations of this work by applying the contraction scheme: (C,N)[4*s*3*p*2*d*1*f*]/(H)[3*s*2*p*1*d*].

The one-dimensional potential energy surfaces for the dissociation reaction of **PDP** (Figure 4) are built with an interpolation method that is explained in detail in other publications.^58−63^

The geometries and molecular orbitals of the chemical species have been analyzed with the programs Gabedit,^64^ Molden,^65^ and MacMolPlt.^66^

## Supporting Information Available

Dissociation and isomerization potential energy curves calculated with the CASSCF and MS-CASPT2 methods. Cartesian coordinates of the featuring species of this work (PDF)
