# Supplementary material for: Photochemistry of 1-Phenyl-1-diazopropane and Its Diazirine Isomer: A CASSCF and MS-CASPT2 Study
Source: J Phys Chem A. 2022 Nov 6;126(45):8372–9. doi: 10.1021/acs.jpca.2c04816 (PMC9677432; doi:10.1021/acs.jpca.2c04816)
Supplement: Supplementary file 1 — jp2c04816_si_001.pdf [file jp2c04816_si_001.pdf]

Supporting Information

Photochemistry of 1-phenyl-1-diazopropane and its diazirine isomer: A CASSCF and MS-CASPT2 study

by

Juan Soto

[a]Department of Physical Chemistry, Faculty of Science, University of Málaga,

29071, Málaga, Spain

E-mail: soto@uma.es

CONTENTS

Figure S1 SA2-CASSCF/ANO-RCC potential energy curves connecting S_1 minimum (M1) of 1-phenyl-1-diazopropane with the S_1/S_0 conical intersection CI1a. S3

Figure S2 MS-CASPT2/ANO-RCC potential energy curves of the low-lying singlet and triplet states of PDP leading to dissociation into carbene (EPC) and dinitrogen. Reference wave function: SA2-CASSCF(14e, 14o). A' singlet states (solid blue lines); A" singlet states (dotted blue lines); A' triplet states (solid red lines); A" triplet states (dotted red lines). S4

Cartesian Coordinates S5

Linear Interpolations

The construction of the potential energy curves has been done with a linear interpolation method[53-62] using the full space using the full space of non-redundant internal coordinates, which are built as follows: a common set of 3N-6 internal coordinates is defined for the target geometries, the reactants (R1) and the products (R2). For the dissociation case, the fragments are separated by a physically reasonable distance [Δ(R2-R1)]. Our calculations show that a value of ~4.7 Å for the dissociative bond (in this work, the C-N_2) is enough to reach the asymptotic limit of the potential energy surface (PES) with respect to dissociation of the diazo compound into carbene and N_2. This leads to an excellent agreement between experimental and calculated enthalpies of dissociation.[43,44] Difference between R2 and R1 yields an interpolation vector (ΔR) that connects reactants and products. Afterwards, ΔR is divided in *n* segments. This parameter is chosen in order to ensure a smooth convergence of the CASSCF wavefunction upon a geometrical distortion following the ΔR hyperline. Consequently, each of the divisions constitutes an interpolation step corresponding to a given nuclear conformation on ΔR. The *m*-th one is given by $R_m = R1 + (m/n)$ with $m=1,\ldots,n$. Given that our interpolation vectors result from combination of valence coordinates (internuclear distances, valence bond and dihedral angles), we cannot give a unique unit for them and, in what follows, we will describe them as arbitrary units. Linear interpolations in internal coordinates present two main features that make them particularly appealing to these studies: (i) they are less expensive than scans with relaxation of geometry; (ii) all the points along the interpolation vector (reaction coordinate) are necessarily in a straight line within the set of defined coordinates hence providing a suitable set of coordinates for the representation of reduced dimensionality potential energy surfaces.

Thus, in accordance with the linear interpolation method, we have determined the potential energy curves for the dissociation processes of the electronic states of the diazo compound. In consequence, these potential energy curves have provided reasonable guesses geometries for subsequent optimizations of the critical points related to the sought dissociation channels in the ground state and in the S_1/S_0 surface crossing.

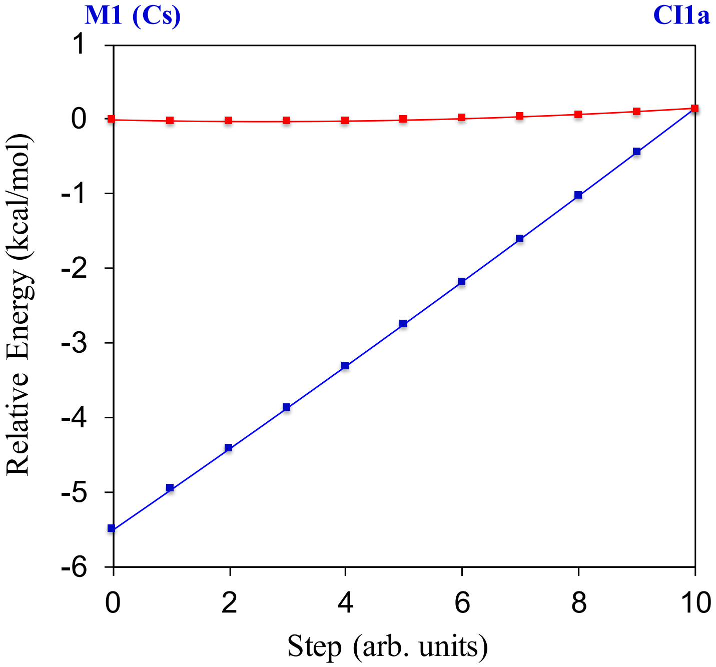

Figure S1. SA2-CASSCF(14e, 14o)/ANO-RCC potential energy curves connecting S_1 minimum (M1) of 1-phenyl-1-diazopropane with the S_1/S_0 conical intersection CI1a.

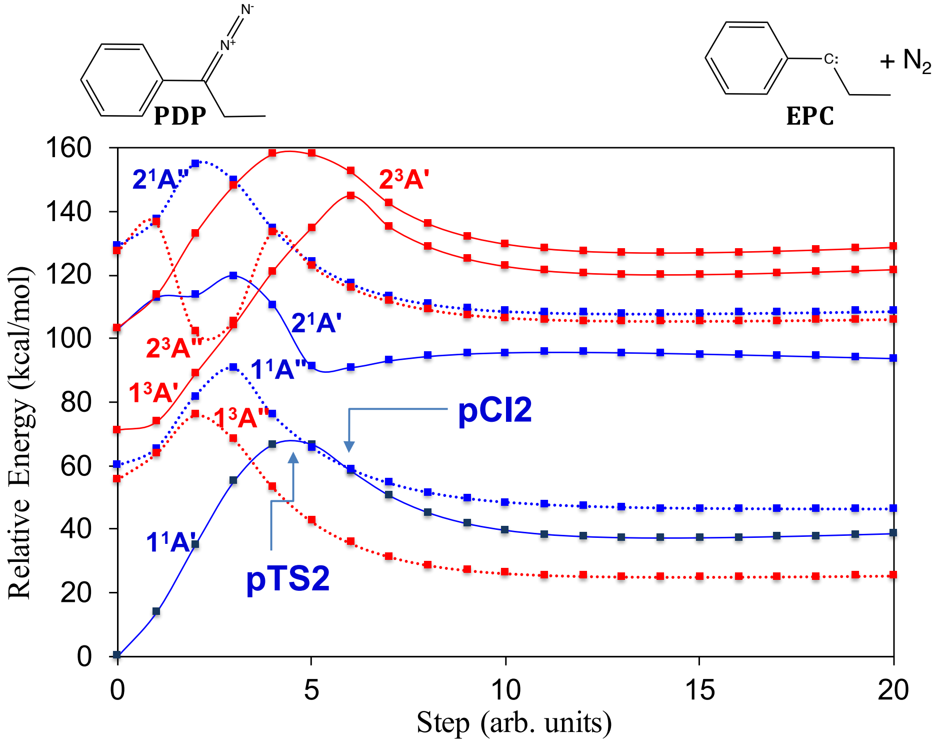

Figure S2. MS-CASPT2/ANO-RCC potential energy curves of the low-lying singlet states of 1-leading to dissociation into phenyl radical and nitrogen dioxide. Reference wave function: SA2-CASSCF(14e, 14o). A' singlet states (solid blue lines); A" singlet states (dotted blue lines); A' triplet states (solid red lines); A" triplet states (dotted red lines). pTS2 and pCI2 are, respectively, the initial structures to start the optimizations of TS2 and CI2 given in the main text.

Cartesian Coordinates in Å of the relevant species in the photochemistry of 1-phenyl-1-diazopropane

PDP (C_s) -ground state- CASSCF(14e,14o)/ANO-RCC (Figure 3a)

C	6	0.000000000000	-0.032650377045	2.453521885698
C	6	0.000000000000	0.018003061030	0.987794838475
C	6	0.000000000000	1.232212896059	0.283229167686
C	6	0.000000000000	-1.168141737946	0.245464568858
C	6	0.000000000000	1.253274870776	-1.103220832490
C	6	0.000000000000	-1.142434466467	-1.145611799413
C	6	0.000000000000	0.065394053311	-1.829601888642
H	1	0.000000000000	2.164456772710	0.813419782285
H	1	0.000000000000	-2.115849667886	0.744431669039
H	1	0.000000000000	2.196379652914	-1.614864593057
H	1	0.000000000000	-2.068233092687	-1.688120144134
H	1	0.000000000000	0.084149810598	-2.901847454289
C	6	0.000000000000	-1.311401903908	3.259111179996
C	6	0.000000000000	-1.110807385695	4.770908314009
H	1	-0.868544804145	-1.900791447538	2.982449158694
H	1	0.868544804145	-1.900791447538	2.982449158694
H	1	0.000000000000	-2.072231301070	5.267491217217
H	1	0.877038615663	-0.567801196423	5.101960383057
H	1	-0.877038615663	-0.567801196423	5.101960383057
N	7	0.000000000000	1.096265725185	3.094570071112
N	7	0.000000000000	2.100262231658	3.642686889054

PDP (C_1) -ground state- CASSCF(14e,14o)/ANO-RCC (Figure 3b)

C	6	0.010601873281	0.037074218470	0.006046610717
C	6	0.000639118970	0.014079757669	1.474210407494
C	6	1.189646120618	0.007265771322	2.219697128726
C	6	-1.210275799956	-0.010937863716	2.174620413244
C	6	1.163212664657	-0.026327445907	3.605936288858
C	6	-1.232767817656	-0.043831006061	3.564877826989
C	6	-0.048804789525	-0.052105272572	4.290076415679
H	1	2.138862459382	0.027551575485	1.721302375945
H	1	-2.140712452042	-0.004863481783	1.644497649737
H	1	2.088049955953	-0.031527801851	4.149674229557
H	1	-2.176595640964	-0.062873243971	4.074840349482
H	1	-0.067300953679	-0.077132763110	5.361977968566
C	6	-1.206233013690	0.129261430603	-0.884233434915
C	6	-1.792161056257	1.537610833596	-1.003418623880
H	1	-0.933623991772	-0.227580128174	-1.869939754139
H	1	-1.962602384297	-0.555191879118	-0.520272475211
H	1	-2.670332047476	1.530779828912	-1.638717167490
H	1	-2.079807948080	1.931546581987	-0.037067206873
H	1	-1.069604871228	2.218874861552	-1.436371633908
N	7	1.159166174152	0.019084429202	-0.591401311031
N	7	2.179764739913	-0.006895373790	-1.123068933082

M1 -S$_1$ state- CASSCF(14e,14o)/ANO-RCC (Figure 3c)

C	6	0.000000000000	-0.005537515860	2.480815984759
C	6	0.000000000000	0.061442571267	1.056570513562
C	6	0.000000000000	1.272862378092	0.316184795267
C	6	0.000000000000	-1.147773673198	0.306270868129
C	6	0.000000000000	1.263801590322	-1.068031558830
C	6	0.000000000000	-1.140596930636	-1.075234516826
C	6	0.000000000000	0.064690242437	-1.779056667289
H	1	0.000000000000	2.209958528555	0.824356973945
H	1	0.000000000000	-2.090321845663	0.810706061422
H	1	0.000000000000	2.198021091576	-1.595244102131
H	1	0.000000000000	-2.072668613050	-1.606397404440
H	1	0.000000000000	0.067783923062	-2.851180368735
C	6	0.000000000000	-1.324039219974	3.223028104998
C	6	0.000000000000	-1.227241499184	4.744953911519
H	1	-0.866359223224	-1.897889694166	2.910346599913
H	1	0.866359223224	-1.897889694166	2.910346599913
H	1	0.000000000000	-2.225954029142	5.164156163883
H	1	0.873734825259	-0.707281381357	5.114005994424
H	1	-0.873734825259	-0.707281381357	5.114005994424
N	7	0.000000000000	1.126144394139	3.325995867070
N	7	0.000000000000	2.294656185651	3.061525187225

PED -ground state- CASSCF(14e,14o)/ANO-RCC (Figure 3f)

C	6	0.030645817037	0.000000000000	-0.012382199772
C	6	0.016542823201	0.000000000000	1.479280988038
C	6	1.211743017631	0.000000000000	2.207744377361
C	6	-1.187717259399	0.000000000000	2.187977934091
C	6	1.199454217622	0.000000000000	3.596951316296
C	6	-1.198542715309	0.000000000000	3.578825417379
C	6	-0.005676139101	0.000000000000	4.290163258938
H	1	2.153809056815	0.000000000000	1.698906467395
H	1	-2.122900588001	0.000000000000	1.667169365406
H	1	2.128776422675	0.000000000000	4.132771407155
H	1	-2.136555744525	0.000000000000	4.099477899978
H	1	-0.014084536138	0.000000000000	5.362706058087
C	6	-1.267126489601	0.000000000000	-0.788111468027
C	6	-1.111248454833	0.000000000000	-2.305439905360
H	1	-1.843471838163	-0.869639994423	-0.490683144437
H	1	-1.843471838163	0.869639994423	-0.490683144437
H	1	-2.088936174183	0.000000000000	-2.770029489598
H	1	-0.577387483541	0.875482660275	-2.652535730658
H	1	-0.577387483541	-0.875482660275	-2.652535730658
N	7	1.226970936209	0.620501766806	-0.658823329854
N	7	1.226970936209	-0.620501766806	-0.658823329854

C1a -S_1/S_0 conical intersection- SA2-CASSCF(14e,14o)/ANO-RCC (Figure 3d)

C	6	0.061422039195	0.017284046897	0.033133473430
C	6	0.052920337645	0.005768882684	1.453642668339
C	6	1.221849612870	-0.021299469413	2.264989748477
C	6	-1.198487273421	0.018825291162	2.138641792519
C	6	1.133317300166	-0.034214841602	3.645604417197
C	6	-1.268955685601	0.006751368818	3.517226204389
C	6	-0.104582413788	-0.020188551990	4.288474490674
H	1	2.186230776422	-0.033642832876	1.812664653852
H	1	-2.111215838393	0.041748709933	1.582876089597
H	1	2.035854781479	-0.056134046227	4.224994079448
H	1	-2.229280955171	0.018841166476	3.995266397023
H	1	-0.162035391676	-0.030070940171	5.358965802386
C	6	-1.217412806606	0.026280772484	-0.775911183477
C	6	-1.051852973539	-0.023122722753	-2.291225683783
H	1	-1.825757538509	-0.816721191392	-0.466380864695
H	1	-1.785302296233	0.914332871275	-0.515948267256
H	1	-2.032139169557	-0.025937061355	-2.752412521493
H	1	-0.505167685567	0.831272567593	-2.665019230809
H	1	-0.527331502320	-0.914143240651	-2.608654315184
N	7	1.245642829505	0.040900534246	-0.782567533249
N	7	2.385353721831	0.055308720409	-0.403305464478

C1b -S_1/S_0 conical intersection- SA2-CASSCF(14e,14o)/ANO-RCC (Figure 3e)

C	6	0.048557822559	0.536452256442	-0.078117657000
C	6	-0.011699577151	0.224325064400	1.305779492161
C	6	1.153745315391	0.208369315598	2.119760810756
C	6	-1.244419287314	-0.092922974722	1.932616789813
C	6	1.078628621891	-0.099512817513	3.463958033325
C	6	-1.303826828554	-0.401870341395	3.279828205310
C	6	-0.147370538730	-0.407616675842	4.059931074913
H	1	2.101103612710	0.443643054802	1.683119827729
H	1	-2.149116912002	-0.105448068195	1.361463213942
H	1	1.974421783025	-0.100140421589	4.054186857174
H	1	-2.250805176694	-0.640311205718	3.723977682625
H	1	-0.198649919265	-0.646260744182	5.103820805798
C	6	-1.122505872741	0.603542391245	-1.021644182726
C	6	-1.390766397476	-0.718275930527	-1.748356784225
H	1	-2.006476687631	0.911001711830	-0.481825830565
H	1	-0.923470712527	1.377785682495	-1.750812695282
H	1	-2.225538556303	-0.609464412498	-2.430008365635
H	1	-0.527843073726	-1.027449785011	-2.323984023546
H	1	-1.628086993816	-1.509054948713	-1.047217196675
N	7	1.323720713552	0.763767700799	-0.611673197836
N	7	1.623877389753	0.976638197058	-1.750587265825

TS1 -ground state- CASSCF(14e,14o)/ANO-RCC (Figure 3g)

C	6	-0.064392977185	-0.090476403642	0.037859628457
C	6	-0.077736077955	-0.114115312226	1.509456768782
C	6	1.137564438763	-0.133304925966	2.215384520334
C	6	-1.262889230741	0.012775489507	2.243266000717
C	6	1.161269219316	-0.053691703271	3.597513683414
C	6	-1.236137220732	0.120662498048	3.631091385169
C	6	-0.028269541847	0.079061396784	4.313743111354
H	1	2.058218352014	-0.211444400939	1.671885704197
H	1	-2.208359270602	0.034511704680	1.740550633094
H	1	2.100167387241	-0.078516593054	4.115847645215
H	1	-2.156838283665	0.224617106674	4.171996788865
H	1	-0.008996857881	0.152421338633	5.383667557943
C	6	-1.341318409025	-0.065890559044	-0.760921246437
C	6	-1.146546003425	-0.055847533854	-2.273852438789
H	1	-2.051658217227	-0.835622842813	-0.478743966465
H	1	-1.788679568173	0.881079951983	-0.468940155796
H	1	-2.091743083138	0.131348203570	-2.768668858779
H	1	-0.448644964031	0.717830289300	-2.567743996452
H	1	-0.769478088243	-1.002848080974	-2.636879067478
N	7	1.163863266771	-1.233666863139	-0.642100507053
N	7	0.459747449459	-2.078984188626	-0.297899513490

TS2 -ground state- CASSCF(14e,14o)/ANO-RCC (Figure 3h)

C	6	0.025629630610	0.659521848957	0.034376854472
C	6	0.019428135039	0.286985065862	1.372043650258
C	6	1.199191084295	0.337040639617	2.182499686070
C	6	-1.184483219832	-0.155189088481	2.010840144653
C	6	1.163793420681	-0.020479959252	3.513290042024
C	6	-1.196429568154	-0.510163953767	3.341600998982
C	6	-0.027362214741	-0.448727653249	4.111922262187
H	1	2.122551690936	0.663229322134	1.751525597418
H	1	-2.091993854888	-0.218774975291	1.446815045097
H	1	2.063453898237	0.031534379522	4.095361394047
H	1	-2.113335827808	-0.840257190899	3.790443203204
H	1	-0.045000852649	-0.727607752239	5.146601305298
C	6	-1.113683251722	0.704037800727	-0.938798058196
C	6	-1.280586612603	-0.573502868320	-1.766870786940
H	1	-2.030449337647	0.914494011002	-0.405483742754
H	1	-0.950034092057	1.544723138258	-1.603826540235
H	1	-2.106018609886	-0.464793867776	-2.460052587653
H	1	-0.388122240200	-0.790116673632	-2.339414045454
H	1	-1.482590863979	-1.425839368586	-1.130006286494
N	7	1.334841116918	1.120400165817	-0.575747404864
N	7	1.966603503155	0.248982903546	-1.103114870061

CI2 -S_1/S_0 conical intersection- SA2-CASSCF(14e,14o)/ANO-RCC (Figure 3i)

C	6	-0.348557163816	-0.000036529097	0.094102933885
C	6	-0.086854927040	0.000005805073	1.490484350503
C	6	1.135896297823	-0.000026607026	2.161003235762
C	6	-1.259862263477	0.000029295246	2.281443389456
C	6	1.188100187845	-0.000030655231	3.554591983582
C	6	-1.188476316365	0.000026416522	3.664158415119
C	6	0.037603850691	-0.000007276186	4.321452841120
H	1	2.058800187559	-0.000036370344	1.620717810700
H	1	-2.223972906389	0.000047408979	1.809462751212
H	1	2.150858486853	-0.000054875668	4.029735524111
H	1	-2.102769562856	0.000048610211	4.226920194239
H	1	0.087685253582	-0.000016124027	5.392263225475
C	6	-1.285280540742	-0.000013462266	-1.047551202695
C	6	-0.666979346444	-0.000025866178	-2.445103566589
H	1	-1.927744597775	-0.877664192990	-0.980795809469
H	1	-1.927710005466	0.877663430975	-0.980795693050
H	1	-1.466344358804	-0.000023416088	-3.173184927067
H	1	-0.061655961945	0.880327340394	-2.612589974831
H	1	-0.061669244291	-0.880389756837	-2.612573453921
N	7	1.657449656859	-0.000038381217	-0.778094256098
N	7	2.679459358103	0.000215205755	-1.190232258374

Cartesian Coordinates in Å of the relevant species in the chemistry of 1-ethyl-1-phenyl carbene

EPC-C_s -S_0 ground state- CASSCF(10e,10o)/ANO-RCC (Figure 4a)

C	6	-0.042100364010	0.313319264070	0.016439167985
C	6	-0.019046968753	0.157447817556	1.474645491637
C	6	1.248411256699	0.085950177027	2.068806958824
C	6	-1.148659325208	0.097075253027	2.314613085061
C	6	1.393216820966	-0.075940638165	3.442731616814
C	6	-1.005971792401	-0.025486244775	3.687189897152
C	6	0.265809238685	-0.123886483249	4.252902801869
H	1	2.108779115299	0.154756353883	1.433755779063
H	1	-2.133468456360	0.161811295745	1.898047013235
H	1	2.371765818429	-0.145266424629	3.876409096791
H	1	-1.874803045418	-0.055263544557	4.315875425305
H	1	0.371044577675	-0.231082534630	5.315302683061
C	6	-1.357165575972	0.071665850063	-0.648370790359
C	6	-1.256940755923	-0.111769253049	-2.158434255428
H	1	-1.925568161035	-0.766980679116	-0.197617579354
H	1	-1.934047742980	0.974051648044	-0.428020475921
H	1	-2.239028387308	-0.216922162117	-2.605792006675
H	1	-0.759469523068	0.735182054994	-2.612551000935
H	1	-0.683001406432	-0.998661739538	-2.399174452587

TSH1 -S_0 ground state- CASSCF(10e,10o)/ANO-RCC (Figure 4b)

C	6	0.148925001839	-0.102188188909	-0.043982202550
C	6	0.077656600842	-0.130432938807	1.437275717158
C	6	1.199600921398	-0.226121345581	2.264005606019
C	6	-1.188454159718	-0.126010737832	2.031786187245
C	6	1.060929673665	-0.292816005149	3.646548469195
C	6	-1.324737781411	-0.180728965358	3.413495887490
C	6	-0.200612764240	-0.264359748444	4.229201683222
H	1	2.182859870483	-0.262536712066	1.832999401607
H	1	-2.063963802735	-0.091307666827	1.411375264276
H	1	1.936358082701	-0.368076232653	4.262767727205
H	1	-2.305212623783	-0.171877816400	3.849710417253
H	1	-0.306686092255	-0.318386791262	5.295161746429
C	6	0.577944839352	1.090378814834	-0.633245193471
C	6	0.770631932515	2.456444652694	-0.015410442354
H	1	1.441538148974	0.162975787060	-0.470364515416
H	1	0.591563165148	1.092226992417	-1.712645419117
H	1	0.836227454142	2.417222879973	1.060295798841
H	1	1.660766475604	2.931144769248	-0.409301274190
H	1	-0.075150571420	3.078682993627	-0.286385571676

E-EPA -S_0 ground state- CASSCF(10e,10o)/ANO-RCC (Figure 4c)

C	6	-0.059297467106	-0.317590601937	0.069040517657
C	6	-0.040060529055	-0.127374739509	1.532033999875
C	6	1.183312595354	0.106928848681	2.163886002259
C	6	-1.192919562714	-0.166908451565	2.327390135640
C	6	1.262666494796	0.285766355058	3.541889901526
C	6	-1.117211503189	0.011189016473	3.700997419647
C	6	0.112067312067	0.237247104221	4.317129379306
H	1	2.080115495244	0.146835302523	1.574920101962
H	1	-2.151974680171	-0.327574438665	1.877151724021
H	1	2.215977151093	0.461702377945	4.001774206775
H	1	-2.013677687672	-0.021308486591	4.289888250876
H	1	0.167015964482	0.376246251269	5.379366099313
C	6	-1.086027936353	-0.720645887343	-0.687804313309
C	6	-1.022412055783	-0.890420388699	-2.177173851646
H	1	0.892496017195	-0.109932659154	-0.436859723686
H	1	-2.031649691296	-0.950598708178	-0.230772251620
H	1	-1.745796715233	-0.249985032735	-2.670923212414
H	1	-0.040556923661	-0.648660350792	-2.563002172491
H	1	-1.254840388396	-1.911551777619	-2.460626834799

EPC-C_1 -S_0 ground state- CASSCF(10e,10o)/ANO-RCC (Figure 4d)

C	6	0.412342730324	0.200477892865	0.068137296965
C	6	0.158766537546	0.130583595536	1.502639797876
C	6	1.286233964467	0.074885279049	2.340564968667
C	6	-1.110957560873	0.004825037065	2.092832388967
C	6	1.149414535056	-0.017245125920	3.718320683861
C	6	-1.239903215608	-0.167475216145	3.464875827554
C	6	-0.115245666325	-0.152783052368	4.284587689951
H	1	2.260490128888	0.109873460108	1.894692696999
H	1	-1.990353015051	0.002812131933	1.483785539558
H	1	2.021710866374	-0.023381717916	4.342750950548
H	1	-2.215154666957	-0.291043383476	3.894796790217
H	1	-0.222163936754	-0.260249926020	5.346539924334
C	6	-0.339344772090	1.163006693043	-0.789376132573
C	6	-1.632970441452	1.877771850824	-0.396262053386
H	1	0.468717266389	1.924751423762	-0.916201940771
H	1	-0.431035240361	0.731970658917	-1.780366941612
H	1	-1.558539651472	2.359646265141	0.569322464250
H	1	-1.855020001862	2.640943375377	-1.132931318368
H	1	-2.477838717811	1.200953990275	-0.373854190725

TSH2 -S_0 ground state- CASSCF(10e,10o)/ANO-RCC (Figure 4e)

C	6	0.148925001839	-0.102188188909	-0.043982202550
C	6	0.077656600842	-0.130432938807	1.437275717158
C	6	1.199600921398	-0.226121345581	2.264005606019
C	6	-1.188454159718	-0.126010737832	2.031786187245
C	6	1.060929673665	-0.292816005149	3.646548469195
C	6	-1.324737781411	-0.180728965358	3.413495887490
C	6	-0.200612764240	-0.264359748444	4.229201683222
H	1	2.182859870483	-0.262536712066	1.832999401607
H	1	-2.063963802735	-0.091307666827	1.411375264276
H	1	1.936358082701	-0.368076232653	4.262767727205
H	1	-2.305212623783	-0.171877816400	3.849710417253
H	1	-0.306686092255	-0.318386791262	5.295161746429
C	6	0.577944839352	1.090378814834	-0.633245193471
C	6	0.770631932515	2.456444652694	-0.015410442354
H	1	1.441538148974	0.162975787060	-0.470364515416
H	1	0.591563165148	1.092226992417	-1.712645419117
H	1	0.836227454142	2.417222879973	1.060295798841
H	1	1.660766475604	2.931144769248	-0.409301274190
H	1	-0.075150571420	3.078682993627	-0.286385571676

Z-EPA -S_0 ground state- CASSCF(10e,10o)/ANO-RCC (Figure 4f)

C	6	0.783500689491	0.210299145212	0.101042906724
C	6	0.480050340752	0.007181298776	1.538081330273
C	6	1.437561245261	-0.594024098096	2.361549995739
C	6	-0.750487413353	0.353607291664	2.099876096398
C	6	1.185058763461	-0.816986176839	3.709230775474
C	6	-1.008388464974	0.127881976954	3.448487153651
C	6	-0.040553134753	-0.453492750420	4.259128692506
H	1	2.384390010902	-0.881668306991	1.944723298885
H	1	-1.513745823500	0.782958304100	1.481369523165
H	1	1.936888185890	-1.272539124950	4.324488767353
H	1	-1.962078567732	0.398175351506	3.859444027284
H	1	-0.239690145690	-0.628073555970	5.298712281545
C	6	0.574815010638	1.302772247328	-0.644287326248
C	6	0.016616738662	2.627298093299	-0.208143558051
H	1	1.256061157699	-0.643509081319	-0.400834736461
H	1	0.857552334641	1.242543661313	-1.680734863449
H	1	-0.983594997730	2.777659276575	-0.602413047874
H	1	-0.026778146068	2.722358991249	0.866532343522
H	1	0.633006434042	3.431789826604	-0.592964426186

CI4 -S_1/S_0 conical intersection- SA4-CASSCF(10e,10o)/ANO-RCC (Figure 6e)

C	6	-0.264121862157	0.422077266415	0.137785320550
C	6	-0.091912833792	0.200260332272	1.480735902345
C	6	1.226782686737	0.251851826769	2.060950620535
C	6	-1.187285546113	-0.079220435678	2.381438721259
C	6	1.423960477563	0.042232631834	3.404333481836
C	6	-0.966844779428	-0.280859094451	3.721689547177
C	6	0.335687126231	-0.225548193833	4.254638793901
H	1	2.054023697204	0.459034155732	1.412368201658
H	1	-2.185239415989	-0.124418935985	1.995195223913
H	1	2.416050705005	0.084810303031	3.810696457682
H	1	-1.795915753201	-0.484356985318	4.372005545037
H	1	0.495462502931	-0.386005595184	5.302213788400
C	6	-1.450888682389	0.423289441882	-0.754806901048
C	6	-1.198135465882	-0.298027891862	-2.079304436041
H	1	-2.310083127747	-0.024400690812	-0.259057308940
H	1	-1.715673619364	1.456970388133	-0.960131066855
H	1	-2.080513462904	-0.259079021078	-2.706936822330
H	1	-0.376767273779	0.160231717889	-2.614794293914
H	1	-0.948830039459	-1.338841214464	-1.912262335501

CI5 -S_1/S_0 conical intersection- SA4-CASSCF(10e,10o)/ANO-RCC (Figure 6l)

C	6	0.496337708986	0.944585885421	0.381163169359
C	6	0.244118379284	0.505276082210	1.669030396702
C	6	1.363844548067	0.101657207286	2.476016771538
C	6	-1.054828753500	0.412933688883	2.285057330576
C	6	1.200532495400	-0.348398638223	3.766200953316
C	6	-1.198204766919	-0.038926317255	3.576847586107
C	6	-0.080120597726	-0.425192828514	4.334717708344
H	1	2.340325230261	0.161557893985	2.039781947833
H	1	-1.923425528130	0.698923335523	1.733922128622
H	1	2.057282031293	-0.641935218577	4.341465399381
H	1	-2.177899623370	-0.097510776388	4.011025593563
H	1	-0.205998131091	-0.775406205617	5.340039613021
C	6	-0.273802168176	1.444558146975	-0.785891083127
C	6	-1.801176916889	1.551413170155	-0.723122690313
H	1	0.140686147862	2.443118554384	-1.031758847916
H	1	0.002207308933	0.823019203579	-1.633241935778
H	1	-2.125183463428	2.234056440089	0.052094974785
H	1	-2.172070493870	1.928622836014	-1.669096115209
H	1	-2.263474259264	0.587970777413	-0.549398458490

CI6 -S_1/S_0 conical intersection- SA4-CASSCF(10e,10o)/ANO-RCC (Figure 6g)

C	6	-0.071497555861	-0.153026251593	0.006332091195
C	6	-0.014271542044	-0.106858621584	1.465320094136
C	6	1.209117738142	-0.313308183100	2.124803339621
C	6	-1.132673995405	0.235533358796	2.238703567803
C	6	1.298575925611	-0.221274495835	3.503727673712
C	6	-1.036275051916	0.344799614607	3.622972024680
C	6	0.174841319451	0.111351007321	4.261900210095
H	1	2.078728548447	-0.546094377039	1.541992843568
H	1	-2.075377682446	0.415973097102	1.760075602256
H	1	2.240686616760	-0.390998016266	3.988471519262
H	1	-1.904962056539	0.606092961507	4.196340312672
H	1	0.247967719558	0.194020862950	5.328862047941
C	6	-1.177832045412	-0.379467236036	-0.811564582521
C	6	-1.231909614063	0.041949500903	-2.250673152292
H	1	-0.084995896034	-1.767398519416	-0.226083647003
H	1	-2.023141343282	-0.952687163928	-0.457238197348
H	1	-1.833254512610	-0.638788382109	-2.839218668985

H	1	-1.684554564512	1.027537887711	-2.326436553161
H	1	-0.240177046858	0.100423658355	-2.673162525980

CI7 -S_1/S_0 conical intersection- SA4-CASSCF(10e,10o)/ANO-RCC (Figure 6n)

C	6	-0.040187457481	0.221945497727	0.155894518637
C	6	-0.046421799077	0.160343988109	1.610903527615
C	6	1.178347421602	0.066350064712	2.291803047782
C	6	-1.228002059013	0.091476193867	2.365555797814
C	6	1.218044961605	-0.077414809228	3.673050681141
C	6	-1.181001842875	-0.027540478640	3.746794615082
C	6	0.040222938808	-0.122936065250	4.410826582502
H	1	2.095890122590	0.098035646016	1.735673694337
H	1	-2.177014722791	0.129666007686	1.868191040689
H	1	2.166837006146	-0.146408664046	4.169735413904
H	1	-2.097313778404	-0.041976086762	4.305135622005
H	1	0.071264908428	-0.227427989749	5.477677049731
C	6	0.337886227627	1.243260209480	-0.701217657052
C	6	0.412281107648	2.723459081708	-0.427690454604
H	1	-1.370856785334	1.163427626991	-0.243336578285
H	1	0.529194232273	0.970231814687	-1.728349591820
H	1	0.443569192258	2.931871345322	0.632428233943
H	1	1.306007943219	3.124374861646	-0.893636949545
H	1	-0.437196179796	3.238424598242	-0.862744102040

S_3/S_2 conical intersection- SA4-CASSCF(10e,10o)/ANO-RCC (Figure 6a)

C	6	-0.047956391584	-0.241273120862	0.031993472433
C	6	-0.004760673250	-0.098125584961	1.385985678996
C	6	1.250009138792	0.008176956129	2.099553012594
C	6	-1.226545562466	-0.125057308815	2.198245229841
C	6	1.274936588393	0.093114986644	3.516909190246
C	6	-1.152457822143	-0.013965042651	3.608750976717
C	6	0.080641323896	0.088760631469	4.260476713033
H	1	2.164501789809	0.013926396845	1.543201261877
H	1	-2.177695503876	-0.219622203751	1.717376077441
H	1	2.218540733821	0.168228536831	4.019227426309
H	1	-2.060754597451	-0.014754559052	4.178332356753
H	1	0.116617558032	0.163707903619	5.329039010065
C	6	-0.938935840045	0.044515454344	-1.112126476109
C	6	-0.904051652875	-1.012960370159	-2.212655162288
H	1	-1.976623017005	0.142869617365	-0.724620949519
H	1	-0.692768523959	1.017442960828	-1.528714376491
H	1	-1.579230376683	-0.748231600044	-3.017792819781
H	1	0.093059666467	-1.104911902675	-2.624822777316
H	1	-1.196073797078	-1.981971166829	-1.827572055134

S_3/S_2 conical intersection- SA4-CASSCF(10e,10o)/ANO-RCC (Figure 6b)

C	6	0.131800030878	-0.793920213625	0.082381766307
C	6	0.086492414248	-0.409114247117	1.441074208801
C	6	1.270528357695	-0.289702252835	2.220122957562
C	6	-1.133573083223	-0.080602143622	2.096130935226
C	6	1.228998007413	0.126915725975	3.547175899497
C	6	-1.156524024496	0.343357638680	3.427854394503
C	6	0.017132109560	0.453136492501	4.166645463978
H	1	2.216486452330	-0.525008271844	1.770586457071
H	1	-2.063759582697	-0.169729859973	1.565285310469
H	1	2.146334498657	0.194059836845	4.100890972249
H	1	-2.100253754240	0.578593806308	3.882715475269
H	1	-0.006097708061	0.773016658664	5.189516279289
C	6	-0.905261727508	-1.113373863022	-0.791401330489

C	6	-1.356110587280	-0.296842439840	-1.943916309054
H	1	1.066277098517	-1.255222967585	-0.323393373124
H	1	-1.408023922015	-2.083203667135	-0.753588449540
H	1	-2.431508053620	-0.378329367622	-2.070117645905
H	1	-1.040167498959	0.729273818805	-1.855418842664
H	1	-0.911842574288	-0.730445946146	-2.841335631507

S_2/S_1 conical intersection- SA4-CASSCF(10e,10o)/ANO-RCC (Figure 6c)

C	6	-0.119297162845	-0.622544083603	0.119954930017
C	6	-0.151064724269	-0.013849094650	1.407031185721
C	6	1.193270084353	0.214073315874	2.080844066316
C	6	-1.313468435849	0.082093364772	2.245494884202
C	6	1.309403297142	0.043737548088	3.441289143461
C	6	-1.179973979803	0.060334659571	3.600134888240
C	6	0.162165802087	-0.086125694497	4.235877707800
H	1	2.031216776778	0.479409607792	1.470047714983
H	1	-2.290551141696	0.098794194972	1.801704188981
H	1	2.273904287564	0.097793521020	3.910269728390
H	1	-2.041302363685	0.136590142253	4.233409096492
H	1	0.231096414946	-0.179338129109	5.299559192498
C	6	-0.930338902487	-0.135686307716	-1.033371277087
C	6	-0.849673257610	-1.035870051492	-2.261209328233
H	1	-1.988316386909	-0.037812351772	-0.708633794055
H	1	-0.615168412775	0.874111186617	-1.287876371523
H	1	-1.457505158665	-0.646807384157	-3.069790394224
H	1	0.171476144500	-1.113016668459	-2.614550951811
H	1	-1.195418014321	-2.036016942518	-2.029399053339

S_2/S_1 conical intersection- SA4-CASSCF(10e,10o)/ANO-RCC (Figure 6d)

C	6	-0.043155215116	-0.568873372788	0.100717134699
C	6	-0.010981081612	-0.294742654408	1.465735466432
C	6	1.268326652747	-0.209665778398	2.186647043028
C	6	-1.229961654840	-0.075697994576	2.261299653477
C	6	1.318128221480	0.057487882411	3.537744274978
C	6	-1.166781683930	0.193748728319	3.612057307171
C	6	0.102743507389	0.268192139174	4.298605994280
H	1	2.175657050073	-0.363690894157	1.636596045636
H	1	-2.180484098011	-0.110745120676	1.770609280481
H	1	2.264363197464	0.112562341396	4.038444382631
H	1	-2.070456118181	0.351563868888	4.166867564911
H	1	0.144640471586	0.464420607396	5.349142893715
C	6	-1.256180000298	-0.724582319589	-0.729184102114
C	6	-1.158613819065	-0.530475661151	-2.211078976458
H	1	0.923083323117	-0.691510246581	-0.406242566882
H	1	-2.041295860098	-1.360876795723	-0.362801892455
H	1	-2.139163783423	-0.503883145128	-2.669081993510
H	1	-0.649614054982	0.396387526575	-2.451662224972
H	1	-0.599329665036	-1.336759315219	-2.683207281580

S_1/S_0 conical intersection- SA4-CASSCF(10e,10o)/ANO-RCC (Figure 6f)

C	6	-0.039256925958	-0.192139114370	-0.009447367538
C	6	0.015348549797	-0.116177790671	1.452266627883
C	6	1.176374400551	-0.499368163527	2.146775509584
C	6	-1.057772856917	0.397430248941	2.196109804329
C	6	1.246890983724	-0.397877213112	3.526702396206
C	6	-0.973255299965	0.519315410746	3.580216158376
C	6	0.172250970944	0.114959460874	4.254583166801
H	1	2.018489315388	-0.867243144826	1.592839277834
H	1	-1.947932287206	0.719968260154	1.691356961880

H	1	2.140664307507	-0.704498232455	4.035593932982
H	1	-1.803368043586	0.925226067149	4.126343524317
H	1	0.233918220237	0.204343060826	5.321682056232
C	6	-1.146176812417	-0.457883200767	-0.819616557595
C	6	-1.302905139581	0.039704546308	-2.220517337401
H	1	0.002767617553	-1.391961290017	-0.194227310823
H	1	-1.917373355762	-1.156073022704	-0.504789133416
H	1	-1.176644307345	-0.765641268300	-2.936595484183
H	1	-2.314069826528	0.416428697443	-2.349180268671
H	1	-0.588954544155	0.819267925119	-2.434975650804

S_3/S_2 conical intersection- SA4-CASSCF(10e,10o)/ANO-RCC (Figure 6h)

C	6	0.304417425234	0.669805496279	0.309138764748
C	6	0.172215838874	0.360786547817	1.621868411116
C	6	1.344684192530	0.080946283384	2.432598989314
C	6	-1.128584562176	0.227169116297	2.287547034884
C	6	1.217351363850	-0.270657173245	3.805533869110
C	6	-1.200197870242	-0.108154348827	3.667769636269
C	6	-0.045004562181	-0.355161977028	4.411550613234
H	1	2.312657675025	0.144482210582	1.980544214241
H	1	-2.027292727278	0.390916978081	1.733971543183
H	1	2.102051734080	-0.467419009679	4.377410570819
H	1	-2.163300944025	-0.178806930523	4.133383321129
H	1	-0.122722589904	-0.614428199806	5.448862936992
C	6	-0.267477919386	1.368282751358	-0.862587359774
C	6	-1.753726916067	1.727480587722	-0.753020240306
H	1	0.307972495398	2.302917810120	-1.020617383299
H	1	-0.107407918028	0.769860106505	-1.752283252065
H	1	-1.943239491506	2.367672469289	0.098579571312
H	1	-2.065839889119	2.253974804821	-1.646720722857
H	1	-2.367406097397	0.840656306874	-0.658677001795

S_3/S_2 conical intersection- SA4-CASSCF(10e,10o)/ANO-RCC (Figure 6i)

C	6	0.131800030878	-0.793920213625	0.082381766307
C	6	0.086492414248	-0.409114247117	1.441074208801
C	6	1.270528357695	-0.289702252835	2.220122957562
C	6	-1.133573083223	-0.080602143622	2.096130935226
C	6	1.228998007413	0.126915725975	3.547175899497
C	6	-1.156524024496	0.343357638680	3.427854394503
C	6	0.017132109560	0.453136492501	4.166645463978
H	1	2.216486452330	-0.525008271844	1.770586457071
H	1	-2.063759582697	-0.169729859973	1.565285310469
H	1	2.146334498657	0.194059836845	4.100890972249
H	1	-2.100253754240	0.578593806308	3.882715475269
H	1	-0.006097708061	0.773016658664	5.189516279289
C	6	-0.905261727508	-1.113373863022	-0.791401330489
C	6	-1.356110587280	-0.296842439840	-1.943916309054
H	1	1.066277098517	-1.255222967585	-0.323393373124
H	1	-1.408023922015	-2.083203667135	-0.753588449540
H	1	-2.431508053620	-0.378329367622	-2.070117645905
H	1	-1.040167498959	0.729273818805	-1.855418842664
H	1	-0.911842574288	-0.730445946146	-2.841335631507

S_2/S_1 conical intersection- SA4-CASSCF(10e,10o)/ANO-RCC (Figure 6j)

C	6	-0.158595941423	-0.145119239302	0.064894695313
C	6	-0.056655904836	-0.071872245019	1.504090452307
C	6	1.250538813347	-0.347055824799	2.118583513354
C	6	-1.214713262399	0.012604628795	2.406804609988
C	6	1.419330971447	-0.342061244674	3.454753510463

C	6	-1.061872725519	0.036538537753	3.745422850377
C	6	0.290746800862	-0.010644260369	4.358020097316
H	1	2.072126905006	-0.571879215301	1.465846059160
H	1	-2.199158451980	0.012079801502	1.985991680029
H	1	2.377300169809	-0.562430657114	3.883462519914
H	1	-1.918790587366	0.069036331865	4.389801306219
H	1	0.482289490273	0.565993643768	5.242034172436
C	6	-0.749386814885	0.935039873974	-0.787332355248
C	6	-2.202960129269	1.323849631684	-0.505593106937
H	1	-0.109701228828	1.834536174325	-0.666962777908
H	1	-0.657124867065	0.638599190702	-1.825466062454
H	1	-2.315561740901	1.798169883727	0.459687647152
H	1	-2.546357122853	2.022544087515	-1.258443444342
H	1	-2.856566153637	0.459318461436	-0.538703370658

S_2/S_1 conical intersection- SA4-CASSCF(10e,10o)/ANO-RCC (Figure 6k)

C	6	0.615355037673	0.558284321469	0.223821197812
C	6	0.363076741764	0.271840847231	1.561979429815
C	6	1.415187371647	-0.304138130837	2.408656253681
C	6	-0.942474548052	0.484260246447	2.195612229657
C	6	1.188545776155	-0.596310095668	3.745704060396
C	6	-1.151550420953	0.178285823345	3.534057979470
C	6	-0.094430161147	-0.354916412381	4.344207817519
H	1	2.377321924281	-0.491035295941	1.974860523783
H	1	-1.756403779742	0.842548453784	1.601200932656
H	1	1.978274975709	-1.007148856965	4.342873428595
H	1	-2.117775581589	0.338083160896	3.969927824417
H	1	-0.260422183759	-0.573234448513	5.378539101296
C	6	-0.287827875693	1.213915713089	-0.730875389262
C	6	-0.821185647160	2.602491751174	-0.520497724748
H	1	1.594495485830	0.242890783596	-0.162767926284
H	1	-0.192348806099	0.896638500431	-1.753937274559
H	1	-1.056725837633	2.797811029587	0.516613300030
H	1	-0.090925569189	3.348527297883	-0.828215823532
H	1	-1.716081763101	2.767224279301	-1.109424661732

S_1/S_0 conical intersection- SA4-CASSCF(10e,10o)/ANO-RCC (Figure 6m)

C	6	0.480708674143	1.364905622296	0.718081637852
C	6	0.282756686595	0.582840553314	1.942132082528
C	6	1.370985692882	-0.032102419420	2.586723944922
C	6	-0.980838371185	0.484030472445	2.542723078294
C	6	1.194096881283	-0.738201105769	3.765075081347
C	6	-1.150259355760	-0.209853869116	3.738369723593
C	6	-0.070260921827	-0.830682646222	4.352220453566
H	1	2.351023688952	0.056185122403	2.158674168463
H	1	-1.826284826476	0.959306524219	2.084292290325
H	1	2.038006542689	-1.206814013136	4.234381533552
H	1	-2.126394464122	-0.266865349233	4.181197073018
H	1	-0.202916362243	-1.370141216144	5.269720550503
C	6	-0.391810033280	1.512274123694	-0.363577009947
C	6	-0.420262532827	2.709233900872	-1.259066989628
H	1	0.825435246431	0.509300855286	-0.072935345996
H	1	-1.017311100471	0.688367077311	-0.698038027478
H	1	0.067923636407	3.550499041908	-0.792121027261
H	1	0.071216128880	2.496304507198	-2.202870555457
H	1	-1.451711251194	2.957431696062	-1.492646197829
